# Screening of four lysosome-related genes in sepsis based on RNA sequencing technology

**DOI:** 10.1186/s12865-023-00588-7

**Published:** 2023-12-06

**Authors:** Guihong Chen, Wen Zhang, Chenglin Wang, Muhu Chen, Yingchun Hu, Zheng Wang

**Affiliations:** 1 Department of Hepatobiliary Surgery, The First Affiliated Hospital of Xi’an Jiaotong University, Xi’an, Shaanxi, China; 2 Department of Emergency Medicine, The Affiliated Hospital of Southwest Medical University, Luzhou, Sichuan China; 3 Department of Endocrinology and Metabolism, The Traditional Chinese Medicine Hospital of Luzhou City, Luzhou, Sichuan China

**Keywords:** RNA sequencing, Lysosome-related genes, Sepsis, Prognosis

## Abstract

**Purpose:**

Screening of lysosome-related genes in sepsis patients to provide direction for lysosome-targeted therapy.

**Methods:**

Peripheral blood samples were obtained from 22 patients diagnosed with sepsis and 10 normal controls for the purpose of RNA sequencing and subsequent analysis of differential gene expression. Concurrently, lysosome-related genes were acquired from the Gene Ontology database. The intersecting genes between the differential genes and lysosome-related genes were then subjected to PPI, GO and KEGG analyses. Core genes were identified through survival analysis, and their expression trends in different groups were determined using meta-analysis. Single-cell RNA sequencing was used to clarify the cellular localization of core genes.

**Results:**

The intersection of 1328 sepsis-differential genes with 878 lysosome-related genes yielded 76 genes. PPI analysis showed that intersecting genes were mainly involved in Cellular process, Response to stimulus, Immune system process, Signal transduction, Lysosome. GO and KEGG analysis showed that intersecting genes were mainly involved in leukocyte mediated immunity, cell activation involved in immune response, lytic vacuole, lysosome. Survival analysis screened four genes positively correlated with sepsis prognosis, namely GNLY, GZMB, PRF1 and RASGRP1. The meta-analysis revealed that the expression levels of these four genes were significantly higher in the normal control group compared to the sepsis group, which aligns with the findings from RNA sequencing data. Furthermore, single-cell RNA sequencing demonstrated that T cells and NK cells exhibited high expression levels of GNLY, GZMB, PRF1, and RASGRP1.

**Conclusion:**

GNLY, GZMB, PRF1, and RASGRP1, which are lysosome-related genes, are closely linked to the prognosis of sepsis and could potentially serve as novel research targets for sepsis, offering valuable insights for the development of lysosome-targeted therapy. The clinical trial registration number is ChiCTR1900021261, and the registration date is February 4, 2019.

## Introduction

Sepsis is a life-threatening organ dysfunction caused by a malfunctioning host response to infection. It is characterized by an acute cytokine storm followed by long-term dysfunction of the immune system in survivors [1]. Due to its high mortality rate and high healthcare costs, sepsis remains one of the most difficult diseases to treat. Overactivation of the immune system and a cascade of inflammation responses in the early stages of sepsis are often accompanied by immunosuppression. The core pathogenesis of sepsis is dysregulation of host innate and adaptive immune responses [2]. Early treatment of sepsis includes source control, antimicrobial, and resuscitation [3]. Despite significant advances have been made in the pathogenesis of sepsis, there is currently no clinically effective treatment [4].

Lysosomes are membrane-enclosed vesicle organelle found in all eukaryotic cells and contain two classes of proteins necessary for maintaining structure and function: soluble lysosomal hydrolases, which perform digestive functions, and lysosomal membrane proteins, which have more complex functions. Lysosomes are organelles responsible for degradation, nutrient sensing, and immunity. Recently, more and more studies have shown that lysosomes are also important in the pathological of many diseases [5]. Existing studies have proved that lysosomes are closely related to atherosclerosis [6], neurodegenerative diseases [7], tumor [8] and other diseases. The body's immunity is affected by lysosomal activity in cells such as macrophages and T cells. For innate immunity, pathogens such as bacteria are internalized by phagocytosis and degraded by targeting lysosomes [9, 10]. In addition, several toll-like receptors (TLRs) on the lysosomal membrane are capable of recognizing various micro-organisms and host-derived ligands and elicting pro-inflammatory signaling [11]. For adaptive immunity, lysosomes produce antigenic peptides for presentation to CD4 + T cells by major histocompatibility complex class II (MHC-II) molecules. In this process, TLR4 signal-induced tubes of phagosomes and lysosomes are important for antigen presentation [12, 13]. It can be seen that lysosomes are widely involved in the process of innate and adaptive immune response, and targeted lysosomal therapy may regulate the immune disorders caused by sepsis, improve the prognosis of sepsis patients, and is expected to become a new treatment direction for sepsis. However, in the field of sepsis treatment, therapeutic research targeting lysosomes is lacking.

Therefore, we intend to use RNA sequencing technology and bioinformatics analysis to screen out lysosome-related genes that contribute to the prognosis of patients with sepsis, so as to provide direction for the treatment of lysosomes.

## Methods

### Volunteer recruitment and blood collection

From January 2019 to December 2020, 22 sepsis patients in the ICU or EICU of the Affiliated Hospital of Southwest Medical University and 10 normal controls in the same period were included. A sample of peripheral blood was collected within 24 h of the patient's admission. The sepsis group was selected as follows: (1) patients with sepsis hospitalized in ICU or EICU; (2) comply with the Sepsis 3.0 standard published in 2016 by the Society of Critical Care Medicine (SCCM) and European Soeiety of Intensive Care Medicine (ESICM) [14]; (3) the patient or his or her legal representative was willing to enter the experiment and signed the informed consent form. The exclusion criteria were: (1) pregnant or lactating women; (2) suffering from mental illness; (3) immunodeficiency or HIV positive. The inclusion criteria for the normal control group were as follows: (1) healthy volunteers with an age > 18 years; (2) no serious heart, liver, kidney, digestive tract, nervous system diseases; (3) the blood pregnancy test of female volunteers of childbearing age was negative; (4) voluntarily participate in the trial and sign the informed consent form. The exclusion criteria were: (1) those with a history of blood sickness, needle sickness, and severe anemia; (2) have any history of serious clinical disease; (3) those who underwent surgery within 4 weeks before the study; (4) those who have a history of substance abuse within 1 year before the study. This study complies with the Declaration of Helsinki and has been approved by the Ethics Committee of the Affiliated Hospital of Southwest Medical University, ethics number: ky2018029, and the clinical trial registration number: ChiCTR1900021261.

### RNA sequencing

RNA sequencing has become a routine method for transcriptomic profiling. Total RNA was extracted from the peripheral blood mixture using the Trizol reagent. The mixture was subjected to centrifugation at 12,000 rpm for 5 min at 4℃, and the resulting supernatant was then transferred to a new EP tube containing 0.3 mL of isoamyl alcohol. After centrifugation at 12,000 rpm for 10 min at 4℃, the upper aqueous phase containing the RNA was transferred to a new tube and mixed with an equal volume of isopropyl alcohol. This mixture was then subjected to centrifugation at 13,600 rpm for 20 min at 4℃. Following the removal of the supernatant, the RNA pellet underwent two washes with 1 mL of 75% ethanol. To dissolve the RNA, 50µL of DEPC-treated water was introduced. The subsequent assessment and quantification of the total RNA were conducted using a Nano Drop and Agilent 2100 bioanalyzer. The sequencing data underwent filtration using SOAPnuke [15]. This involved the removal of reads containing sequencing adapters, reads with an unknown base ('N' base) ratio exceeding 5%. Subsequently, the clean reads were aligned to the reference genome using HISAT2 [16]. Bowtie2 [17] was then utilized to align the clean reads to the reference coding gene set. Finally, the expression level of genes was calculated using RSEM [18].

### Screening for differential expression genes

Due to its intuitive user interface and large annotation database, the iDEP (integrated differential expression and pathway analysis) website is widely used for interactive analysis of RNA sequencing data [19]. Therefore, we used iDEP 9.6 (http://149.165.154.220/idepg/) online platform to quality control and filter the data. Subsequently, boxplot and density distribution analysis were performed on the standardized data to clarify the homogeneity and comparability of the RNA sequencing data. Principal component analysis (PCA) was used to find outlier samples and ensure data stability. The DESeq2 method was adopted for statistical analysis, and the differential genes were screened under the conditions of |Fold Change| (FC) ≥ 4 and False Discovery Rate (FDR)<0.05.

### Screening for lysosome-related genes

The Gene Ontology (GO) knowledgebase (http://geneontology.org/) is a comprehensive resource concerning the functions of genes and gene products (proteins and noncoding RNAs) [20]. Therefore, we used the Gene Ontology database to screen lysosome-related genes. Entered the Gene Ontology database, entered "lysosome" in the search box, selected "Gene Product", limited Organism to "Homo sapiens", and downloaded human lysosome-related genes. Lysosome-related genes closely related to sepsis can be obtained by intersecting differential genes with lysosome-related genes.

### PPI analysis

The functional connections between proteins can often be inferred by associations between the genes that encode them, a group of genes with the same function tends to show similar species coverage, is often located close on the genome, and tends to participate in gene fusion events. The STRING database is a precomputed global resource for the exploration and analysis of these associations [21]. The intersecting genes were submitted to the STRING database, “Homo sapiens” was selected in the species option, “full STRING network” was selected in the network type option, “evidence” was selected in the meaning of network edges, “Text mining, Experiments, Databases” were selected in the active interaction sources, the correlation intensity coefficient was set to 0.15, and points in the network that are not connected were removed.

### GO analysis

To further analyze whether the intersecting genes participate in a specific Biological Process (BP), whether they participate in the formation of Cellular Ccomponents (CC), and the Molecular Function (MF) in which they are expressed. We used the Omicshare website (https://www.omicshare.com/) to perform GO annotation and taxonomic enrichment analysis to determine the main functions of intersecting genes and comprehensively describe the properties of genes and gene products. The calculated pvalue was corrected by FDR and adjusted-pvalue ≤ 0.05 was used as the threshold, and the GO term that met this condition was defined as significantly enriched in differentially expressed genes.

### KEGG analysis

Kyoto Encyclopedia of Genes and Genomes (KEGG) is a knowledge base for systematic analysis of gene functions, linking genomic information with higher-order functional information, which includes metabolic pathway database, hierarchical classification database, gene database, and genomic database, etc. [22]. The Omicshare website was used for KEGG enrichment analysis of intersecting genes to identify the most pronounced pathways in the entire genomic background, with a significant enrichment criterion of adjusted-pvalue≤0.05.

### Survival analysis

Clinical data serves as the fundamental foundation for scientific investigation. To further investigate the correlation between genes and prognosis, the GSE65682 [23] dataset from the GEO public database (https://www.ncbi.nlm.nih.gov/geo/) was employed for survival analysis. This dataset encompasses peripheral blood RNA sequencing data from 479 sepsis patients, along with gene expression values and clinical prognosis information for each individual. Survival analysis was conducted using Graphpad prism 7, with a significance threshold set at a logrank test P-value of less than 0.05.

### Meta-analysis

To enhance the precision of core gene screening and gene expression evaluation across various groups, the peripheral blood RNA sequencing data of sepsis patients from the GEO database were procured for meta-analysis. Specifically, the datasets GSE28750 [24], GSE54514 [25], GSE69528 [26], GSE95233 [27] were utilized. Datasets were homogenized (log2 logarithmic) and categorized into normal control and sepsis, and subsequent meta-analysis was conducted on individual genes within the same grouping across different datasets using R language packages. Random effects model was selected when p≤0.05, and Fixed effect model when p > 0.05, for multiple data heterogeneity tests.

### Single-cell RNA sequencing

In order to enhance the understanding of the cellular distribution of core genes, the peripheral blood samples from two healthy individuals, one individual with systemic inflammatory response syndrome (SIRS), and two individuals with sepsis were subjected and analyzed. The Cell Ranger software was employed for quality control measures, ensuring the acquisition of high-quality cell counts, gene counts, and genome alignment. The utilization of a Unique Molecular Identifier (UMI) and Cell Barcode allowed for the determination of the precise quantity of each transcript molecule within an individual cell. The dimensionality reduction results based on the Mutual Nearest Neighbors (MNN) algorithm were visualized by the t-distributed Stochastic Neighbor Embedding (t-SNE) algorithm, and finally, the optimal cell population classification was obtained. The specific marker gene for each cell population was screened by differentiating the specified cell population from all remaining cell populations using the bimod assay. Based on the HPCA reference dataset [28], cell type annotation was performed using the SingleR package [29].

## Results

### Demographic and clinical characteristics

A total of 22 individuals diagnosed with sepsis were included as participants in this study, comprising 14 males and 8 females. Additionally, there were 10 individuals in the normal control group, consisting of 5 males and 5 females. Among the sepsis group, 12 individuals survived, while 10 individuals died within a 28-day period. Conversely, all 10 individuals in the control group survived without any fatalities. The statistical analysis employed the unpaired t-test to assess the Age, Alanine transaminase (ALT), Aspartate aminotransferase (AST), Direct bilirubin (DBIL), Total bilirubin (TBIL), Creatinine, Urea, Leukocyte count, Neutrophil count, Monocyte count, and Lymphocyte count in both groups. The mean ± standard deviation was used to express the results, which are presented in Table 1.

**Table 1:** Demographic and clinical characteristics

Item	Sepsis(*n* = 22)	NC (*n* = 10)	*P* value
Gender (M/F)	14/8	5/5	-
28-Day Finale (S/D)	12/10	10/0	-
Age (years)	56.09±17.51	53.50±7.663	0.6590
ALT (U/L)	90.02±182.5	20.94±6.443	0.2449
AST (U/L)	146.8±277.8	22.18±4.526	0.1703
DBIL (umol/L)	16.74±14.79	5.24±2.048	0.0214
TBIL (umol/L)	32.30±38.48	16.79±6.368	0.2187
Creatinine (umol/L)	106.5±109.2	63.75±9.259	0.2299
Urea (mmol/L)	10.74±9.923	5.046±1.489	0.0838
Leukocyte (10^9/L)	12.88±7.166	6.877±1.848	0.0147
Neutrophil (10^9/L)	13.67±13.27	4.128±1.151	0.0319
Monocyte (10^9/L)	0.8086±1.084	0.443±0.1832	0.3019
Lymphocyte (10^9/L)	1.145±1.565	2.02±0.5712	0.0985

The statistical analysis employed the unpaired t-test to assess the Gender (M: male, F: female), 28-day Finale (S: survivor, D: decedent), Age, ALT, AST, DBIL, TBIL, Creatinine, Urea, Leukocyte count, Neutrophil count, Monocyte count, and Lymphocyte count. The mean ±standard deviation was used to express the results

### Screening for differential expression genes

**Fig. 1:**
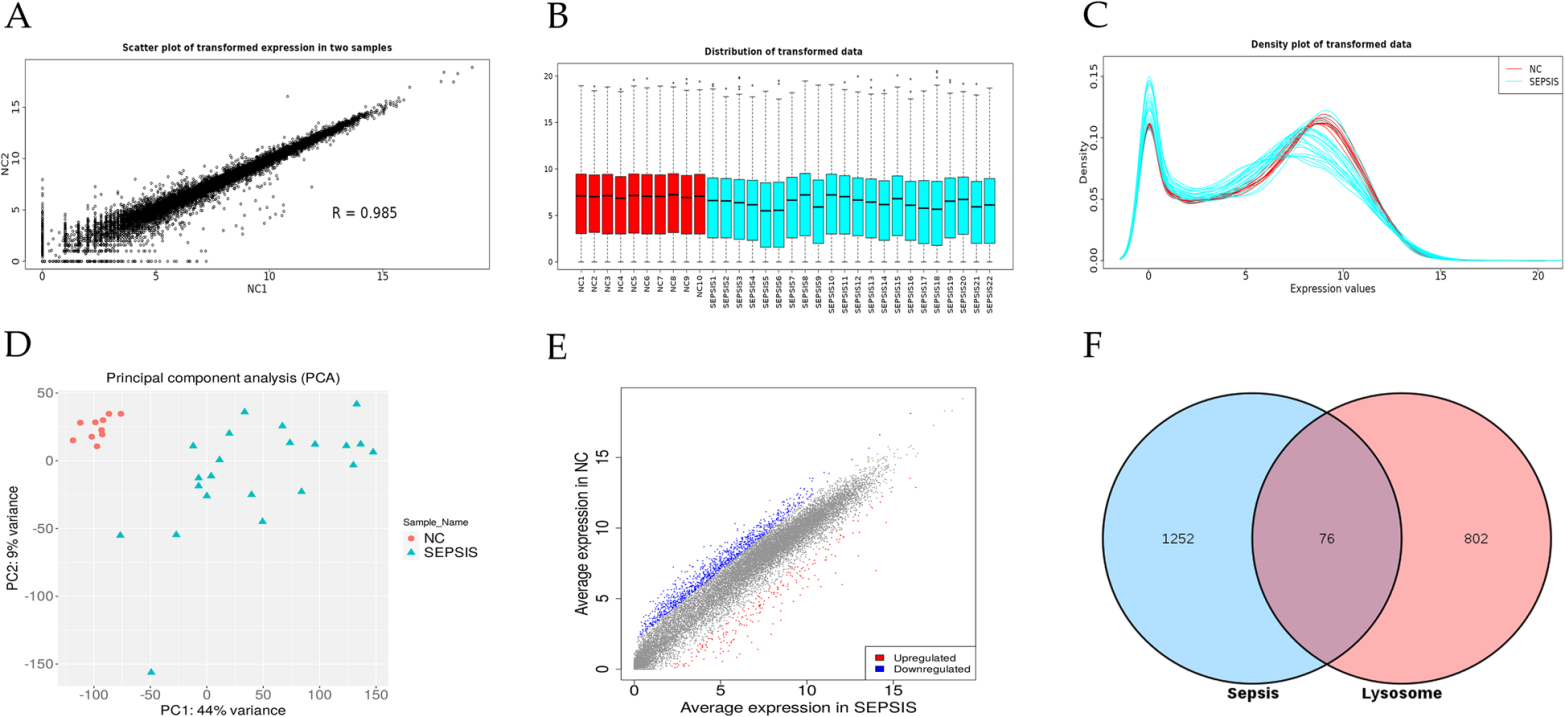
Screening for sepsis-differential genes and lysosome-related genes. **A** Scatter plot with an R value of 0.985 indicates a strong positive correlation and data stability between the two variables. **B**-**C** Box plot, and density distribution plot demonstrated homogeneity and comparability of the two data sets. **D** PCA successfully distinguishes the normal control group from the sepsis group without any outlier samples. **E** The volcano diagram visually represents 221 upregulated genes in the sepsis group, indicated in red, while blue color represents 1107 downregulated genes in the sepsis group. **F** Venn diagram showed the intersection of 1328 sepsis-differential genes with 878 lysosome-related genes, and a total of 76 intersecting genes were obtained

Two representative groups were randomly chosen to generate scatterplots for the purpose of quality control in RNA sequencing data. The obtained Pearson product-moment correlation coefficient (*R*=0.985) indicated a highly robust positive correlation between the two variables, thereby confirming the stability of the data (Fig. 1A). Furthermore, the box plot and density distribution plot demonstrated the homogeneity and comparability of the two datasets (Fig. 1B-C). Additionally, the PCA successfully distinguished the normal group from the sepsis group, with no presence of outlier samples (Fig. 1D). A total of 1328 differential genes were obtained through screening for genes with |FC|≥4 and FDR<0.05. Among these, 221 genes were upregulated in the sepsis group, while 1107 genes were downregulated in the sepsis group (Fig. 1E).

### Screening for lysosome-related genes

A total of 878 genes associated with lysosomes were obtained from the Gene Ontology database for further analysis. 878 lysosome-related genes were found to intersect with 1328 genes that exhibit differential expression in sepsis, resulting in the identification of 76 lysosome-related genes that were closely associated with sepsis (Fig. 1F).

### PPI analysis

**Fig. 2:**
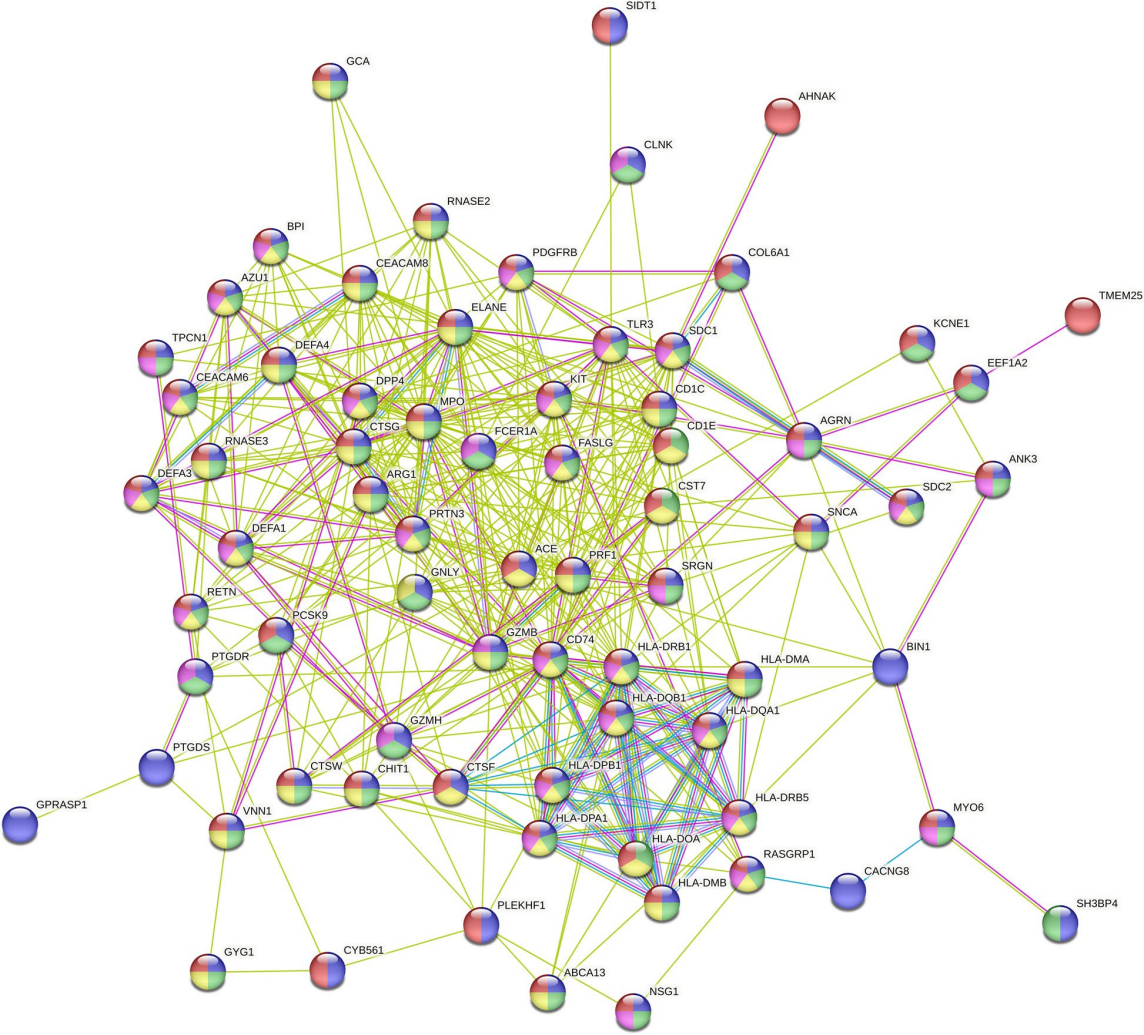
PPI analysis. The PPI network comprises 75 nodes and 450 connections, the dots in the network were color-coded to represent different biological processes, with blue indicating Cellular process, green indicating Response to stimulus, yellow indicating Immune system process, purple indicating Signal transduction, and red indicating Lysosome. The color of the lines connecting the nodes signified the type of interaction evidence, with green denoting Text mining, red denoting Experiments, and blue denoting Databases

The PPI network comprises 75 nodes and 450 connections, with GNLY, GZMB, PRF1, TLR3, KIT, and other proteins occupying central positions within the network. These proteins have the potential to serve as core targets that could impact the prognosis of sepsis. The dots in the network were color-coded to represent different biological processes, with blue indicating Cellular process, green indicating Response to stimulus, yellow indicating Immune system process, purple indicating Signal transduction, and red indicating Lysosome. The color of the lines connecting the nodes signified the type of interaction evidence, with green denoting Text mining, red denoting Experiments, and blue denoting Databases (Fig. 2).

### GO analysis

**Fig. 3:**
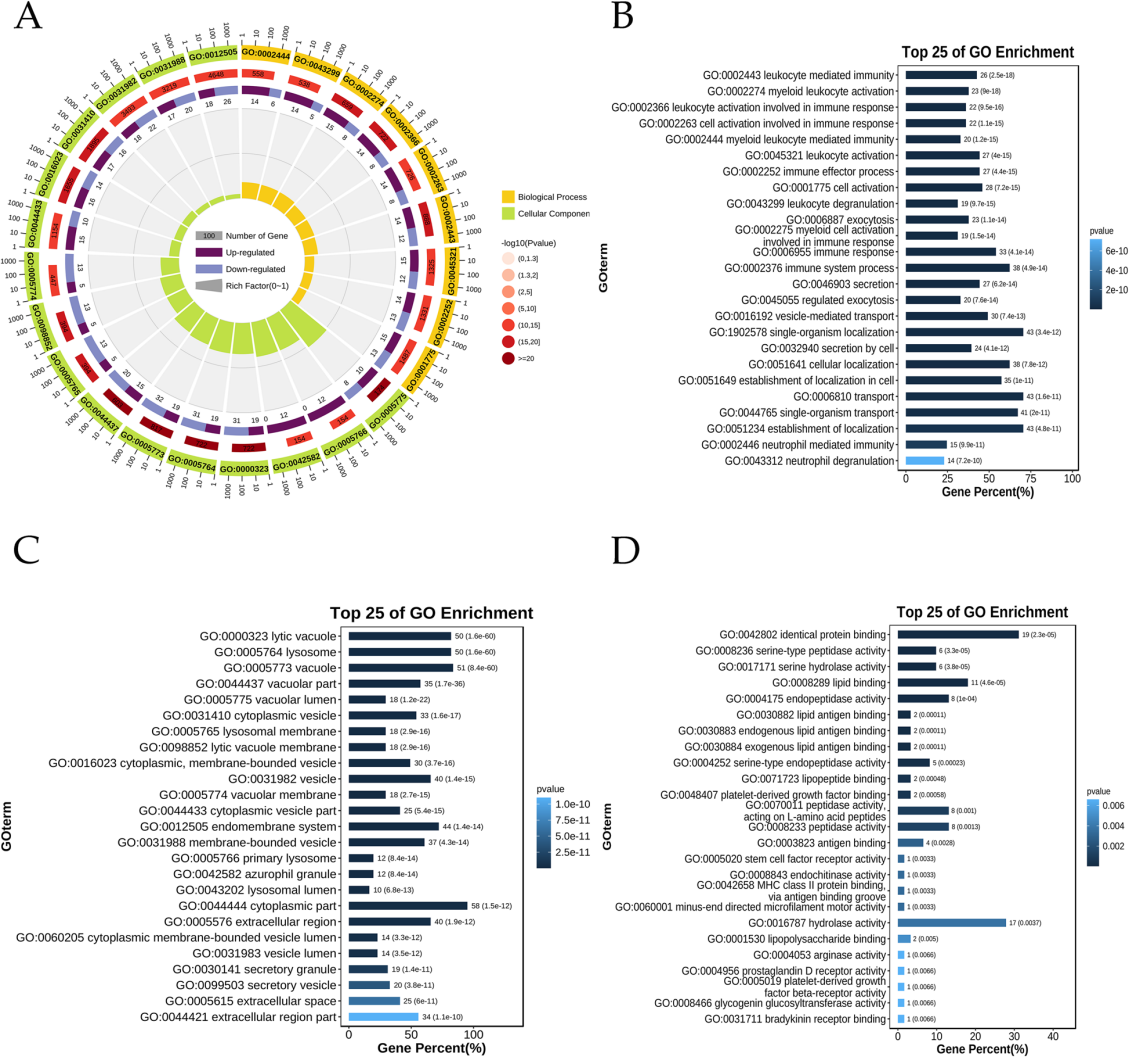
GO analysis. **A** The analysis of GO revealed that among the top 25 items, 9 were related to BPs, 16 were related to CCs, and none were related to MFs. **B** The intersecting genes primarily participated in various BPs, such as leukocyte mediated immunity, cell activation involved in immune response, immune effector process, leukocyte degranulation, and vesicle-mediated transport. **C** The CCs associated with the intersection genes mainly consisted of lytic vacuole, lysosome, cytoplasmic vesicle, lysosomal membrane, and lysosomal lumen. **D** The BPs with the intersection genes mainly involved in identical protein binding, serine-type peptidase activity, serine hydrolase activity, lipid binding, endopeptidase activity

The analysis of GO revealed that among the top 25 items, 9 were related to BPs, 16 were related to CCs, and none were related to MFs (Fig. 3A). Additionally, the taxonomic enrichment analysis indicated that the intersecting genes primarily participated in various BPs, such as leukocyte mediated immunity, cell activation involved in immune response, immune effector process, leukocyte degranulation, and vesicle-mediated transport (Fig. 3B). The CCs associated with the intersection genes mainly consisted of lytic vacuole, lysosome, cytoplasmic vesicle, lysosomal membrane, and lysosomal lumen (Fig. 3C). Furthermore, the MFs with the intersection genes mainly involved in identical protein binding, serine-type peptidase activity, serine hydrolase activity, lipid binding, endopeptidase activity (Fig. 3D).

### KEGG analysis

**Fig. 4:**
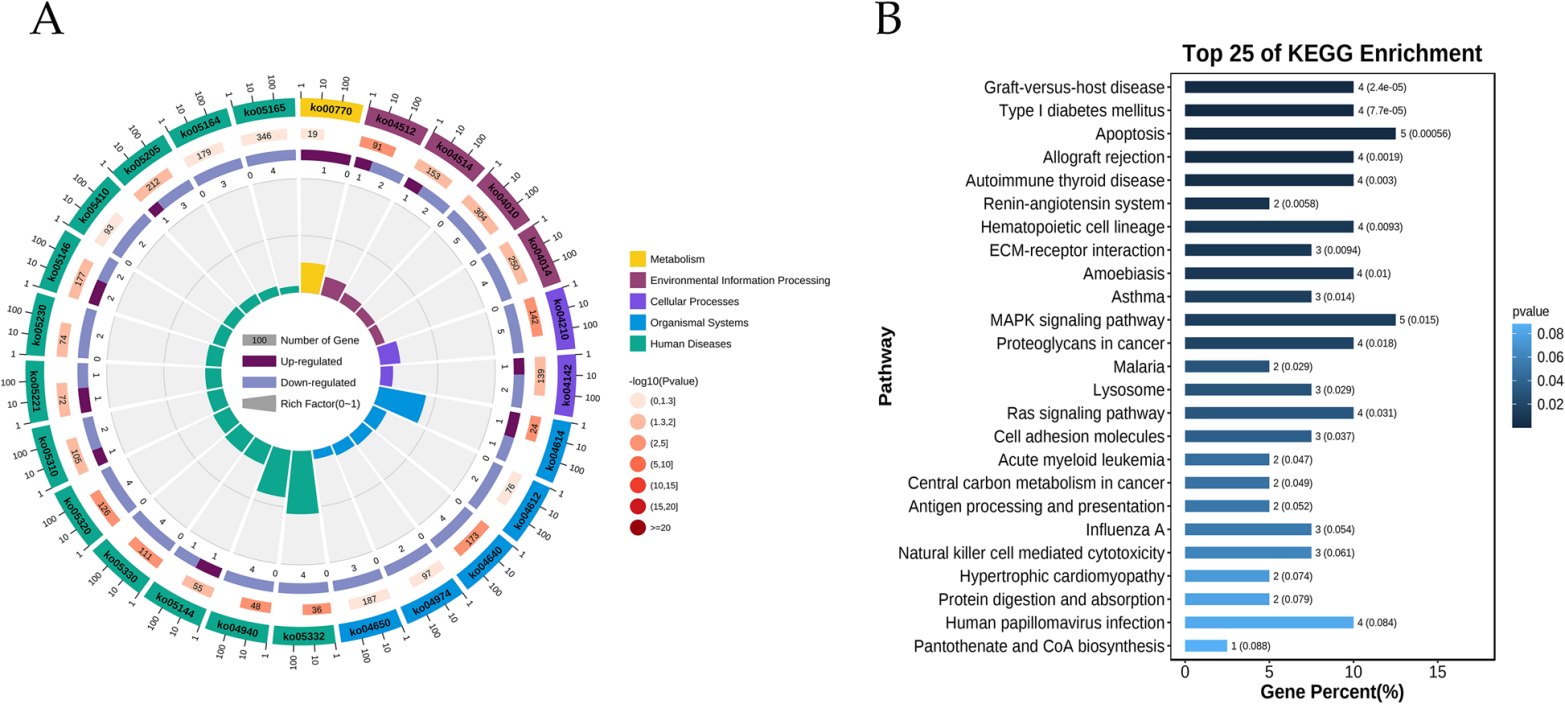
KEGG analysis. **A** The analysis conducted by KEGG identified the 25 most noteworthy items, comprising 1 Metabolism, 4 Environmental Information Processing, 2 Cellular Processes, 5 Organismal Systems, and 13 Human Diseases. **B** Among the pathways with the highest significance for the intersection gene were Graft-versus-host disease, Type I diabetes mellitus, Apoptosis, Allograft rejection, Autoimmune thyroid disease, and Lysosome

The analysis conducted by KEGG identified the 25 most noteworthy items, comprising 1 Metabolism, 4 Environmental Information Processing, 2 Cellular Processes, 5 Organismal Systems, and 13 Human Diseases (Fig. 4A). Among the pathways with the highest significance for the intersection gene were Graft-versus-host disease, Type I diabetes mellitus, Apoptosis, Allograft rejection, Autoimmune thyroid disease, and Lysosome (Fig. 4B).

### Survival analysis

**Fig. 5:**
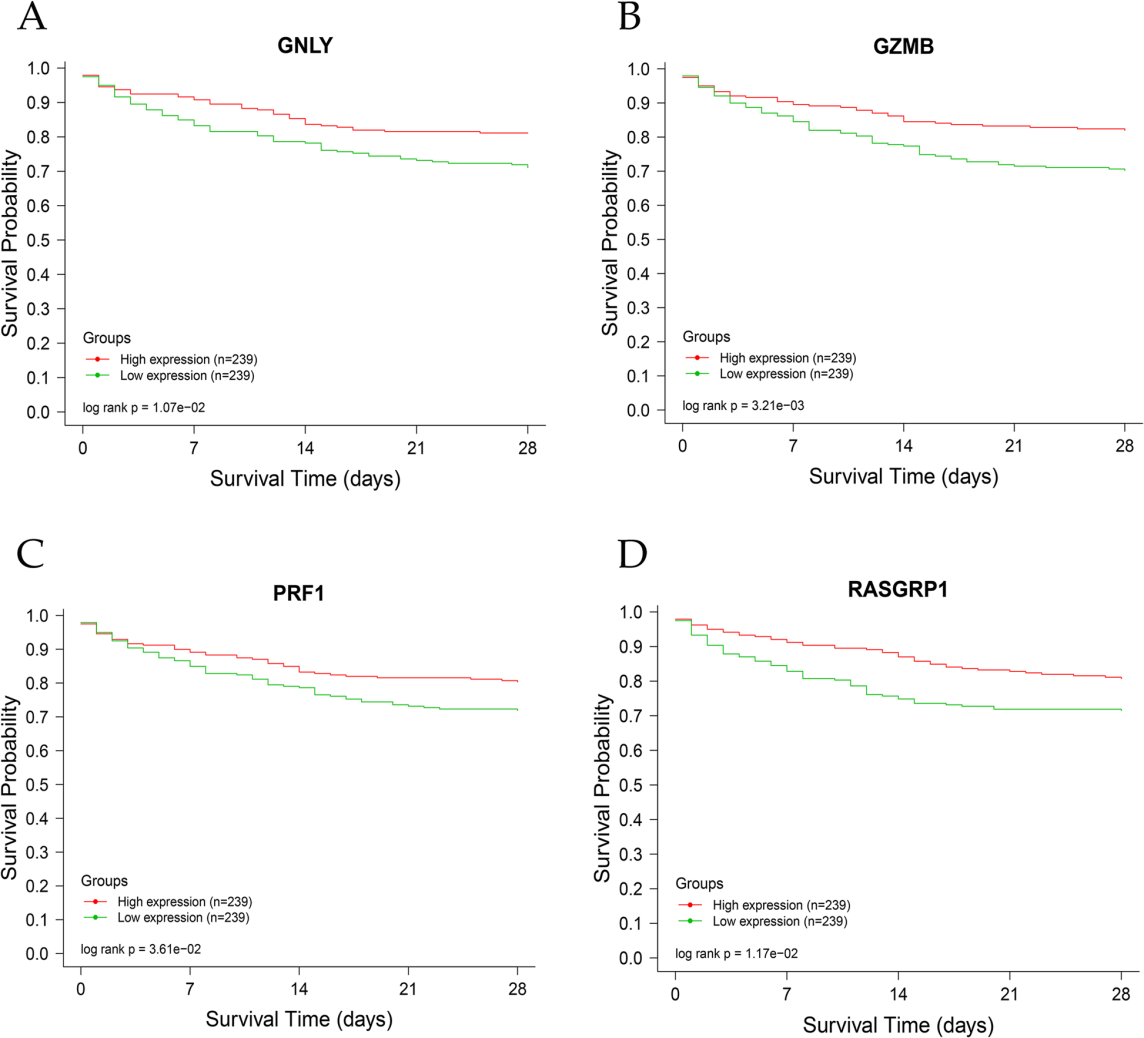
Survival analysis. **A**-**D** Survival analysis based on the GSE65682 dataset observed that patients with high expression levels of GNLY, GZMB, PRF1, and RASGRP1 exhibited a higher 28-day survival rate compared to those with low expression levels (*P*<0.05)

Based on a survival analysis of the GSE65682 dataset in the GEO database, it was observed that patients with high expression levels of GNLY, GZMB, PRF1, and RASGRP1 exhibited a higher 28-day survival rate compared to those with low expression levels (*P*<0.05). This finding suggests a positive correlation between these genes and the prognosis of sepsis patients, with their high expression levels potentially serving as a novel research focus for sepsis. Consequently, these findings provide valuable insights for the development of lysosome-targeting treatments for sepsis (Fig. 5A-D).

### Meta-analysis

**Fig. 6:**
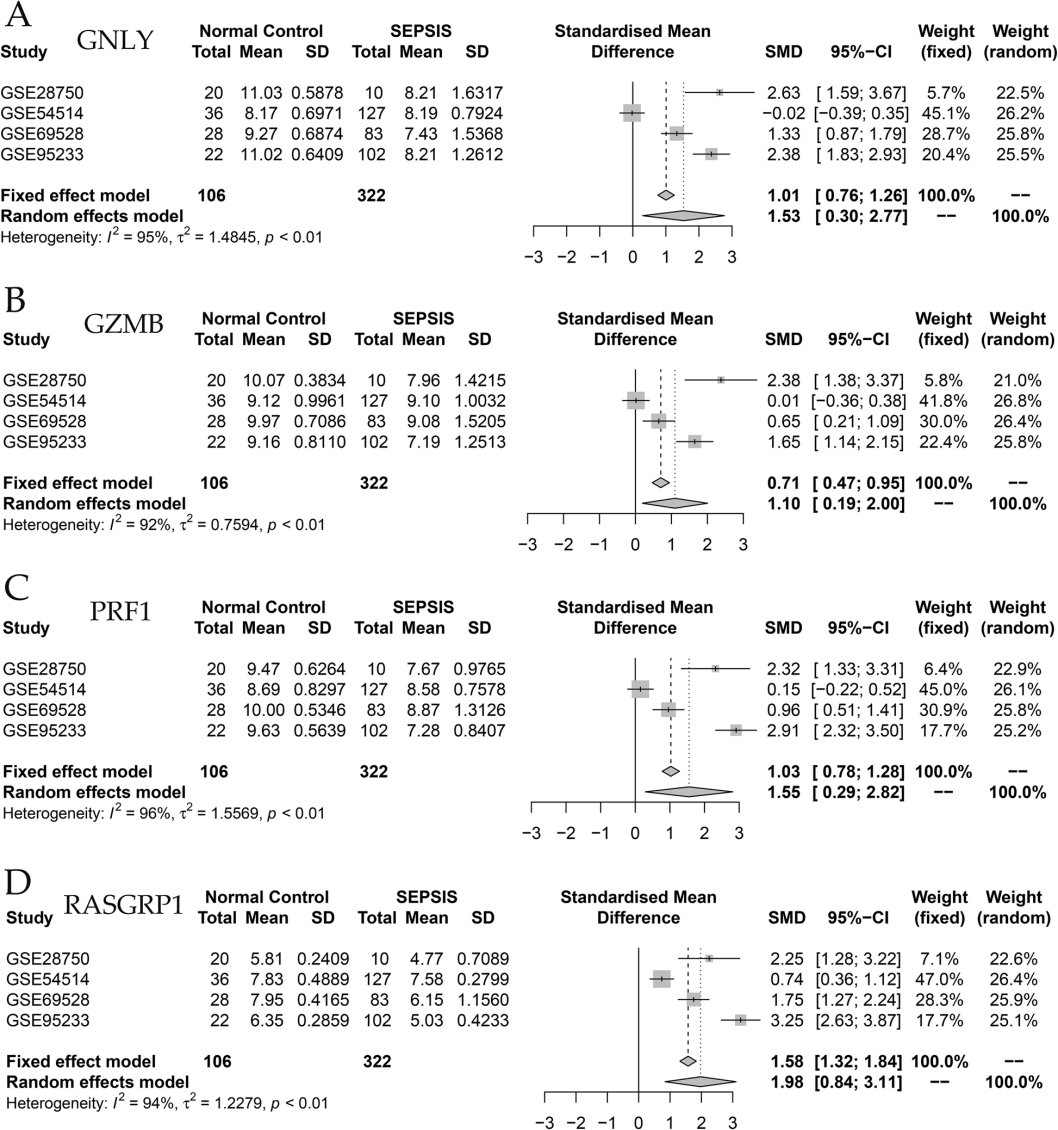
Meta-analysis. **A**-**D** Meta-analysis based on the GSE28750, GSE54514, GSE69528, GSE95233 datasets revealed that GNLY, GZMB, PRF1, and RASGRP1 exhibited high expression levels in the normal control group, while their expression was low in the sepsis group

**Fig. 7:**
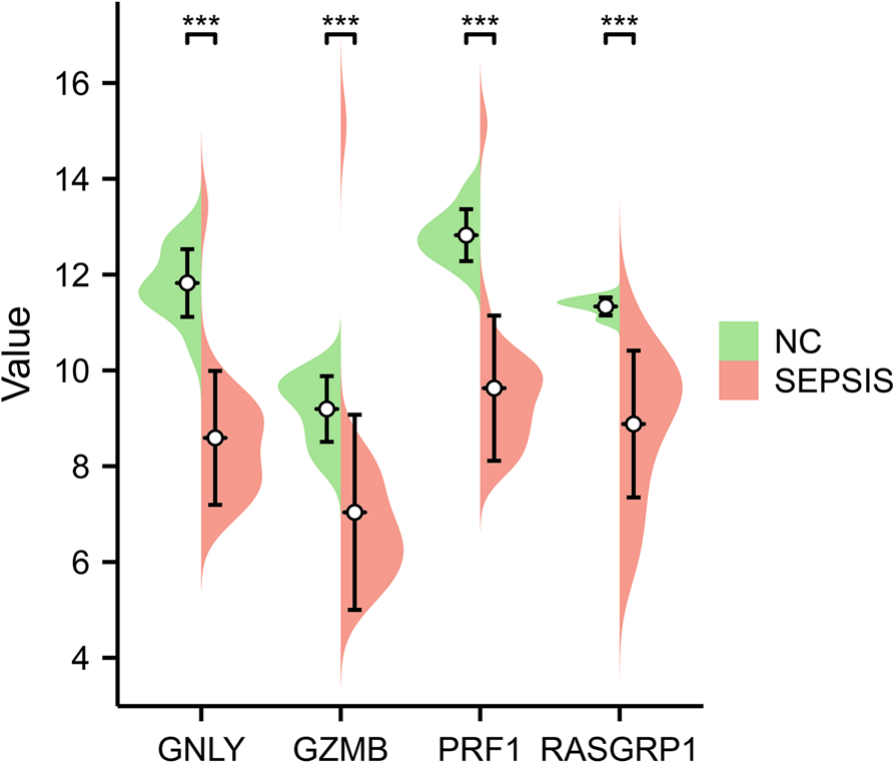
Core gene expression analysis. The pod plot illustrated the expression patterns of core genes as determined through peripheral blood RNA sequencing data obtained from both the normal control group (*n*=10) and the sepsis group (*n*=22). Notably, the analysis revealed that GNLY, GZMB, PRF1, and RASGRP1 exhibited significantly higher expression levels in the normal control group compared to the sepsis group (*P*<0.05). It is worth mentioning that the significance levels were denoted as *P<0.05, **P<0.01, and ***P<0.001

At the transcriptome level, a meta-analysis was conducted on core genes using RNA sequencing datasets GSE28750, GSE54514, GSE69528, and GSE95233. The analysis revealed that GNLY, GZMB, PRF1, and RASGRP1 exhibited high expression levels in the normal control group, while their expression was low in the sepsis group (Fig. 6A-D). Further information regarding the GEO dataset of the included studies can be found in Table 2. Based on the peripheral blood RNA sequencing data of the normal control group (*n*=10) and the sepsis group (*n*=22), the expression of core genes was plotted pod plots, and the results showed that GNLY, GZMB, PRF1 and RASGRP1 were highly expressed in the normal control group and low in the sepsis group, and the difference was statistically significant (Fig. 7).

**Table 2:** The GSE datasets for Survival analysis and Meta-analysis

GSE datasets	Organism	Platform	Number of samples
GSE65682	Whole Blood of Human	GPL13667	802
GSE28750	Whole Blood of Human	GPL570	41
GSE54514	Whole Blood of Human	GPL6947	163
GSE69528	Whole Blood of Human	GPL10558	138
GSE95233	Whole Blood of Human	GPL570	124

The GSE65682 dataset of the GEO database was used for survival analysis, GSE28750, GSE54514, GSE69528, and GSE95233 were used for meta-analysis. Statistics of Organism, Platform, and Number of samples for each dataset were also used

### Single-cell RNA sequencing

**Fig. 8:**
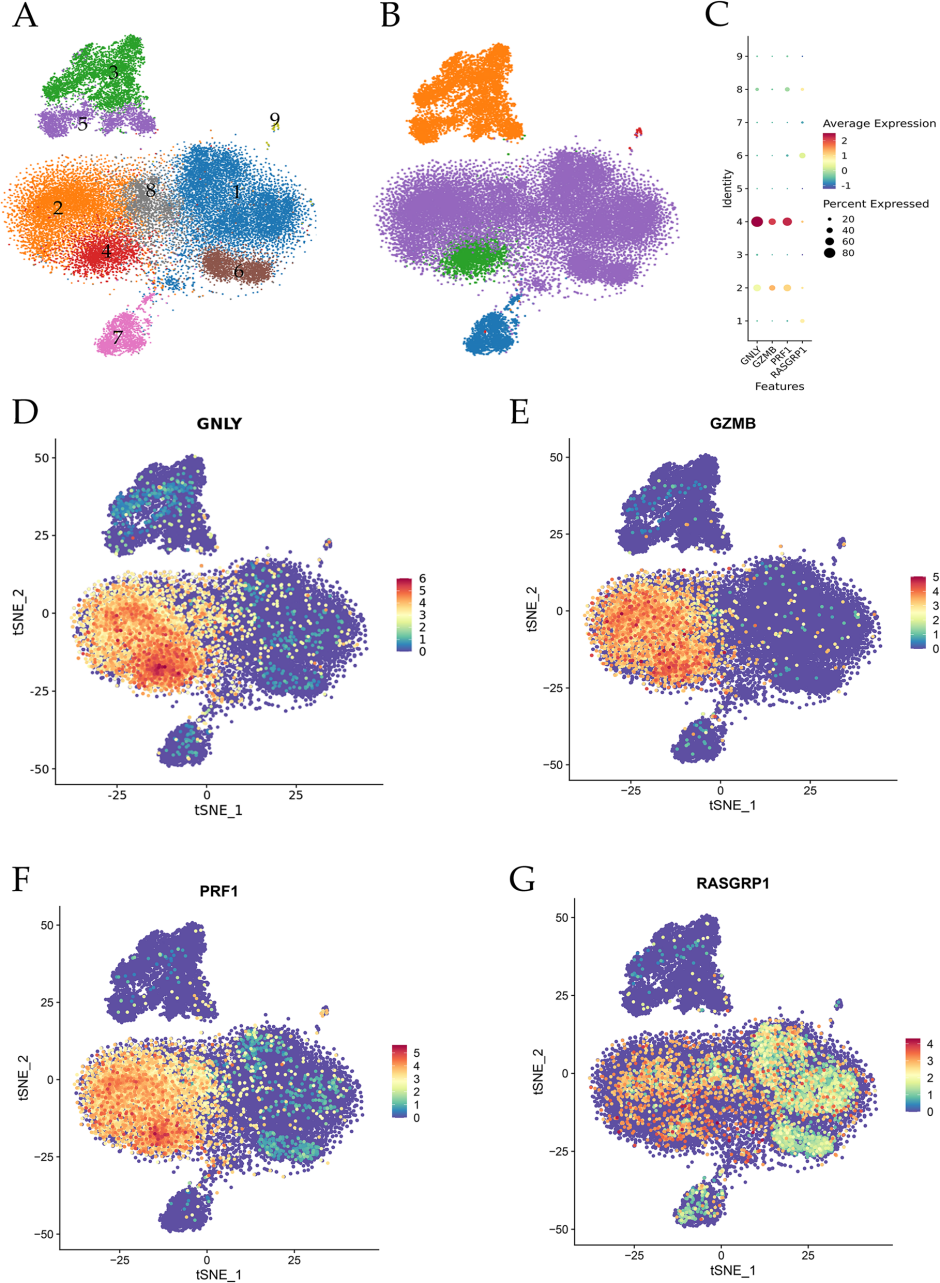
Single-cell RNA sequencing. **A** The total sequencing diagram showed that, following dimensionality reduction, the cells were categorized into nine distinct groups. **B** Among the identified cell lines, T cells were represented by 1, 2, 6, and 8, Macrophages by 3 and 5, NK cells by 4, B cells by 7, and Platelets by 9. **C** In the dotplot, the gene name was plotted on the abscissa, while the cell group number was plotted on the ordinate. The size of the dot corresponded to the proportion of cells expressing the gene within that particular cell group. Additionally, a color gradient ranging from blue to red indicated varying levels of gene expression, with red indicating higher expression. The findings of this study indicate that GNLY, GZMB, and PRF1 were predominantly localized within cell lines 2 and 4, while RASGRP1 exhibited predominant localization within cell lines 1, 2, 4, 6, and 8. **D**-**G**, A dot in a feature plot represented an individual cell, with the color gradient ranging from blue to red, signifying higher gene expression levels within the cell. These results further demonstrate that GNLY, GZMB, PRF1, and RASGRP1 exhibit high expression levels, specifically within T cells and NK cells

The distribution of high-quality cells for quality control of each sample ranged from 4050 to 10,191. After excluding two-cells, multicellular and apoptotic cells, the final number of cells obtained ranged from 3108 to 8509. The average UMI number in each cell ranged from 519 to 8529, and the average gene number in each cell ranged from 343 to 2337. Following dimensionality reduction, the cells were classified into 9 groups (Fig. 8A), with B cells, NK cells, T cells, Platelets, and Macrophages serving as reference cell types. Among the identified cell lines, T cells were represented by 1, 2, 6, and 8, Macrophages by 3 and 5, NK cells by 4, B cells by 7, and Platelets by 9 (Fig. 8B). Analysis of single-cell RNA sequencing data revealed that GNLY, GZMB, and PRF1 were predominantly expressed in cell lines 2 and 4, while RASGRP1 was primarily localized in cell lines 1, 2, 4, 6, and 8 (Fig. 8C). Consequently, GNLY, GZMB, PRF1, and RASGRP1 exhibited high expression levels in T cells and NK cells (Fig. 8D-E).

## Discussion

Deng M. et al. conducted a study which demonstrated that hepatocytes release high mobility group box 1 (HMGB1), which then binds to lipopolysaccharide (LPS) and is internalized into the lysosomes of macrophages and endothelial cells through the receptor for advanced glycation end-products (RAGE). Subsequently, HMGB1 causes permeabilization of the phospholipid bilayer within the acidic environment of the lysosomes. This leads to the leakage of LPS into the cytoplasm and the activation of caspase-11. Caspase-11, being an LPS receptor, plays a role in caspase-11-dependent pyroptosis, which ultimately contributes to the lethality of endotoxemia [30, 31]. Hence, the close association between lysosomes and LPS-mediated sepsis, which can impact the pathophysiological progression of sepsis, is evident. Numerous chemicals exhibit specific affinity for lysosomal proteins, thereby governing cellular behavior [32]. Consequently, this investigation employed RNA sequencing and bioinformatics techniques to identify four lysosome-related genes that significantly influence the prognosis of sepsis patients. These findings offer valuable insights for the development of targeted therapies aimed at lysosomal intervention.

Granulysin (GNLY) is a cytotoxic granule that is co-secreted with granzyme and perforin from the granules of human cytotoxic T lymphocytes (CTLs) and natural killer (NK) cells. It has been documented to possess diverse antimicrobial properties [33, 34]. GNLY is predominantly expressed in human CTLs and NK cells and has been implicated in a plethora of pathological conditions, including infections, cancer, transplantation, and dermatological disorders [35]. Granzyme B (GZMB) is an integral constituent of cytolytic granules found within natural killer (NK) cells and serves as a crucial cytotoxic molecule employed by T cells to eliminate cells infected by pathogens or transformed tumor cells [36, 37]. CD8 + T cells have the ability to induce apoptosis in target cells through the release of GZMB, which, in turn, may lead to tissue damage and restructuring [38]. Emerging evidence indicates its involvement in the lysosome-mediated demise of the cytotoxic lymphocyte itself [39]. Perforin (PRF1) is an essential pore-forming protein involved in lymphocyte cytotoxicity [40]. Mutations in the PRF1 gene have been identified as a causative factor for the development of Hemophagocytic Lymphohistiocytosis (HLH) [41]. HLH is a severe immunodeficiency and multi-organ disorder that can result in a potentially lethal hyperinflammatory state, characterized by fever, hepatosplenomegaly, and distinctive laboratory abnormalities [42]. RAS guanyl-releasing protein 1 (RASGRP1) plays a crucial role as a guanine nucleotide exchange factor and a vital regulator of T cell receptor signaling in the immune system [43]. In murine models, the absence of RASGRP1 leads to impaired development of T lymphocytes [44]. In humans, deficiencies in RASGRP1 can result in a primary immunodeficiency (PID) syndrome, characterized by lymphopenia in CD4 + T cells and the development of Epstein-Barr virus (EBV)-associated B cell lymphoma [45]. By utilizing RNA sequencing and conducting bioinformatics analysis, our study revealed that the genes GNLY, GZMB, PRF1, and RASGRP1 exhibited predominant expression within the lysosomes of T cells and NK cells. These genes were found to actively participate in crucial biological processes, including the immune system process, response to stimulus, cellular process, and signal transduction. Notably, their expression levels were significantly higher in the normal control group compared to the sepsis group. Furthermore, a positive correlation was observed between the expression of these genes and the prognosis of sepsis patients. Previous research has also indicated a strong association between these four genes and the immune response mediated by lymphocytes. Hence, it is postulated that these four genes could serve as innovative targets for precise lysosomal therapy, which plays a significant role in lysosomal-mediated inflammation and immune responses. Consequently, they may modulate the expression of lysosomal proteins, influencing the pathophysiological progression of sepsis and ultimately impacting the diverse clinical outcomes observed in patients.

In conclusion, the genes GNLY, GZMB, PRF1, and RASGRP1 exhibited significant upregulation in the normal control group and downregulation in the sepsis group, thereby displaying a positive correlation with the prognosis of sepsis patients. This observation implies that their heightened expression potentially contributes to the survival of sepsis patients. Additionally, given their association with lysosomal functions, these four genes may serve as promising targets for lysosomal therapy, thereby offering a novel avenue for the clinical management of sepsis.

## Conclusion

GNLY, GZMB, PRF1, and RASGRP1 are lysosome-related genes that are closely related to the prognosis of sepsis, and may be used as new research targets for sepsis and provide direction for lysosome-targeted therapy.

## Data Availability

The datasets generated during the current study are available in the China National GeneBank DataBase (CNGBdb, https://db.cngb.org/) repository, [ACCESSION NUMBER: CNP0002611]. The GEO database analyzed during this study are available in the GEO (https://www.ncbi.nlm.nih.gov/geo/) repository, [ACCESSION NUMBERS: GSE65682, GSE28750, GSE54514, GSE69528, GSE95233].
